# A mouth rinse based on a tea solution of *Salvia officinalis* for oral discomfort in palliative cancer care: a randomized controlled trial

**DOI:** 10.1007/s00520-021-06021-2

**Published:** 2021-02-14

**Authors:** Ragnhild Elisabeth Monsen, Bente Brokstad Herlofson, Caryl Gay, Katrine Gahre Fjeld, Lene Hystad Hove, Karl Egil Malterud, Elisabeth Saghaug, Joran Slaaen, Tone Sundal, Anita Tollisen, Anners Lerdal

**Affiliations:** 1 Department of Medicine, Lovisenberg Diaconal Hospital, Oslo, Norway; 2 Department for Interdisciplinary Health Sciences, Institute of Health and Society, Faculty of Medicine, University of Oslo, Postboks 1089 Blindern, 0317 Oslo, Norway; 3 Department of Oral Surgery and Oral Medicine, Faculty of Dentistry, University of Oslo, Oslo, Norway; 4 Unit of Oral and Maxillofacial Surgery, Department of Otorhinolaryngology – Head and Neck Surgery Division for Head, Neck and Reconstructive Surgery, Oslo University Hospital, Oslo, Norway; 5 Department of Research, Lovisenberg Diaconal Hospital, Oslo, Norway; 6 Department of Family Health Care Nursing, University of California, San Francisco, CA USA; 7 Department of Cariology and Gerodontology, Faculty of Dentistry, University of Oslo, Oslo, Norway; 8 Department of Pharmacy, Section Pharmaceutical Chemistry, University of Oslo, Oslo, Norway

**Keywords:** Palliative care, Oral health, Oral care, Mouth rinse, *Salvia officinalis*, Randomized controlled trial

## Abstract

**Background:**

Few clinical studies evaluate interventions to reduce oral discomfort among patients in palliative care.

**Aim:**

This study examines the efficacy of a *Salvia officinalis* (SO) based herbal mouth rinse compared to conventional normal saline (NS) in order to improve oral health.

**Design:**

A block-randomized controlled trial. Data were collected before and after a 4-day intervention with either SO (*n*=44) or NS (*n*=44). Numerical rating scales (NRS, 0–10) and 12 items from the European Organisation for Research and Treatment of Cancer (EORTC) Quality of Life Questionnaire-Oral Health 17 (EORTC QLQ-OH17) measured patient-reported oral symptoms. An oral examination was performed before and after the intervention.

**Setting/participants:**

This study included adult patients with late-stage cancer in an inpatient hospice unit.

**Results:**

Of the 88 patients included (mean age=63.9 years, SD=10.6), 73 (83%) completed the study. At baseline, 78% reported dry mouth on the EORTC QLQ-OH17, and 80% rated dry mouth ≥4 on the NRS. Total oral health scores based on the 12 EORTC QLQ-OH17 items improved similarly in both groups (*p*<0.001). However, dry mouth ratings on both the EORTC QLQ-OH17 (*p*=0.036) and NRS (*p*=0.045) improved more in the SO group than in the NS group. Plaque on the teeth improved in both the SO (*p*=0.008) and NS (*p*=0.018) groups, but plaque on the tongue and erythema only improved with NS.

**Conclusions:**

This study did not detect an overall significant difference between SO and NS. Both mouth rinses improved oral health parameters, indicating that systematic assessment and oral care may reduce oral discomfort.

**Trial registration:**

NCT02067572

## Introduction

Oral discomfort is highly prevalent in palliative care patients. Moreover, oral symptoms are not limited to patients with head and neck cancer, but are rather common for patients with all types of cancer [1]. Despite its prevalence, poor oral health among cancer patients, particularly those receiving palliative care, is underreported by patients and health staff [2] and is a neglected aspect of patient care [1]. Given that a primary goal of palliative care is to relieve pain and distressing symptoms, research aimed at addressing patients’ oral comfort is needed.

Side effects from cancer and/or cancer treatment often affect patients’ oral health and may cause oral complications, such as xerostomia (subjective experience of dry mouth), salivary gland hypofunction (low saliva secretion), oral infection, dysphagia, mucositis, denture associated stomatitis, oral ulceration, caries, coated mouth and tongue, orofacial pain or dysgeusia (altered taste) [2–5]. Xerostomia is particularly common, with a prevalence of approximately 80% among palliative cancer care patients in Norway, while other common oral symptoms, such as plaque on teeth and tongue, infection, oral ulceration, and pain, have a prevalence of 30–50% [1, 6]. These symptoms may cause nutritional problems, weight loss, and fatigue and can negatively impact social interaction. Saliva lubricates and moistens the oral mucosa, facilitates speech, eating, and swallowing, and plays a significant role in maintaining oral health. Thus, common oral symptoms and discomfort have significant negative impacts on patients’ quality of life [4, 7–10].

Several mouth rinses and products currently exist to treat or manage common oral symptoms, particularly xerostomia [11–14], but there is insufficient evidence to support their long-term or relative effectiveness [15–17]. There is also a multitude of mouth rinses for preventing caries, treating infections, and reducing inflammation [18]. However, a lack of knowledge among nurses and physicians of these products and their effectiveness for treating common oral symptoms may lead to inappropriate guidance and oral hygiene practices.

Moreover, differences in the severity of side effects associated with cancer and its medical treatment may require individual approaches when choosing products. For example, low saliva production can affect taste and sensitivity to the texture of products [9]. This may result in patients preferring a product solely on the basis of its taste or application method, rather than its effectiveness. Thus, an optimal solution for patients would be to introduce a variety of suitable oral health care mouth rinses that are both comfortable to use and proven effective to reduce distressing oral symptoms.

The Norwegian guidelines for palliative care recommend normal saline (NS) (0.9% sodium chloride) rinse as a saliva substitute [6]. However, sage, *Salvia officinalis* L. (SO), has a long history in alternative medicine and has been used to treat dyspepsia, pharyngitis, stomatitis, and inflammation in the mouth or throat [19–21]. It has also been suggested to be beneficial for oral discomfort given its anti-inflammatory, antiseptic, antibacterial, antifungal, and metabolic regulation properties [22–24], and this use has been indicated by the European Medicines Agency [23].

For many years, SO has been used in our hospice unit and reported by our patients to be a pleasant and preferred alternative to NS for relieving oral discomfort. However, SO’s subjective and clinical effects have not been evaluated in a randomized controlled trial (RCT). Therefore, the aim of this study was to investigate the effect of a SO-based herbal mouth rinse compared with NS mouth rinse on perceptions of oral discomfort and symptoms in a RCT among patients receiving palliative care.

## Methods

This study was designed as a prospective, single-blinded, block-randomized controlled trial to evaluate the effect of SO-based mouth rinse on the oral health of patients with advanced cancer in palliative care compared to the current standard of care [6]. It is a sub-study of the project Oral Health in Advanced Cancer (OralHAC) [25]. The overall aim of the OralHAC project is to improve the care of patients with late-stage cancer by addressing their oral health and improving symptom management of oral discomfort. In the current study, data were collected at Lovisenberg Diaconal Hospital in Oslo, Norway, from February 2014 to September 2016. The Regional Committee for Medical Research Ethics, Health Region South-East Norway approved the study in October 2013 (reference #2013/1531). The study was registered with ClinicalTrials.gov (reference #NCT02067572) in February 2014. Randomization was performed from a web-based randomization service in three blocks of 20 subjects and a final block of 28 subjects. The allocation sequence was concealed from the researchers enrolling participants by sequential numbered, opaque sealed envelopes. An analysis was conducted after two blocks (*n*=40) to determine whether there was sufficient beneficial effect from the SO rinse to continue the study.

### Sample

Patients were recruited from a 12-bed inpatient hospice unit, where they were being treated with palliative care. Study inclusion criteria were as follows: (1) admission to the Hospice Lovisenberg inpatient unit, (2) ≥18 years of age and able to provide written informed consent, (3) diagnosis of advanced cancer, and (4) patient report of current oral discomfort. Patients were excluded if they (1) had an estimated life expectancy of ≤2 weeks, (2) currently used antifungal medication, (3) were currently receiving head or neck radiation therapy, (4) were epileptic, (5) had a diagnosis of diabetes, or (6) had any condition that would have interfered with study participation (e.g., inability to use a mouth rinse, anxiety about dental examination or treatment). Exclusion criteria (4) and (5) are related to potential negative interaction with the SO, such as epileptiform convulsion and hypoglycemic activity [26]. Informed written consent was obtained from all study participants.

**Fig. 1:**
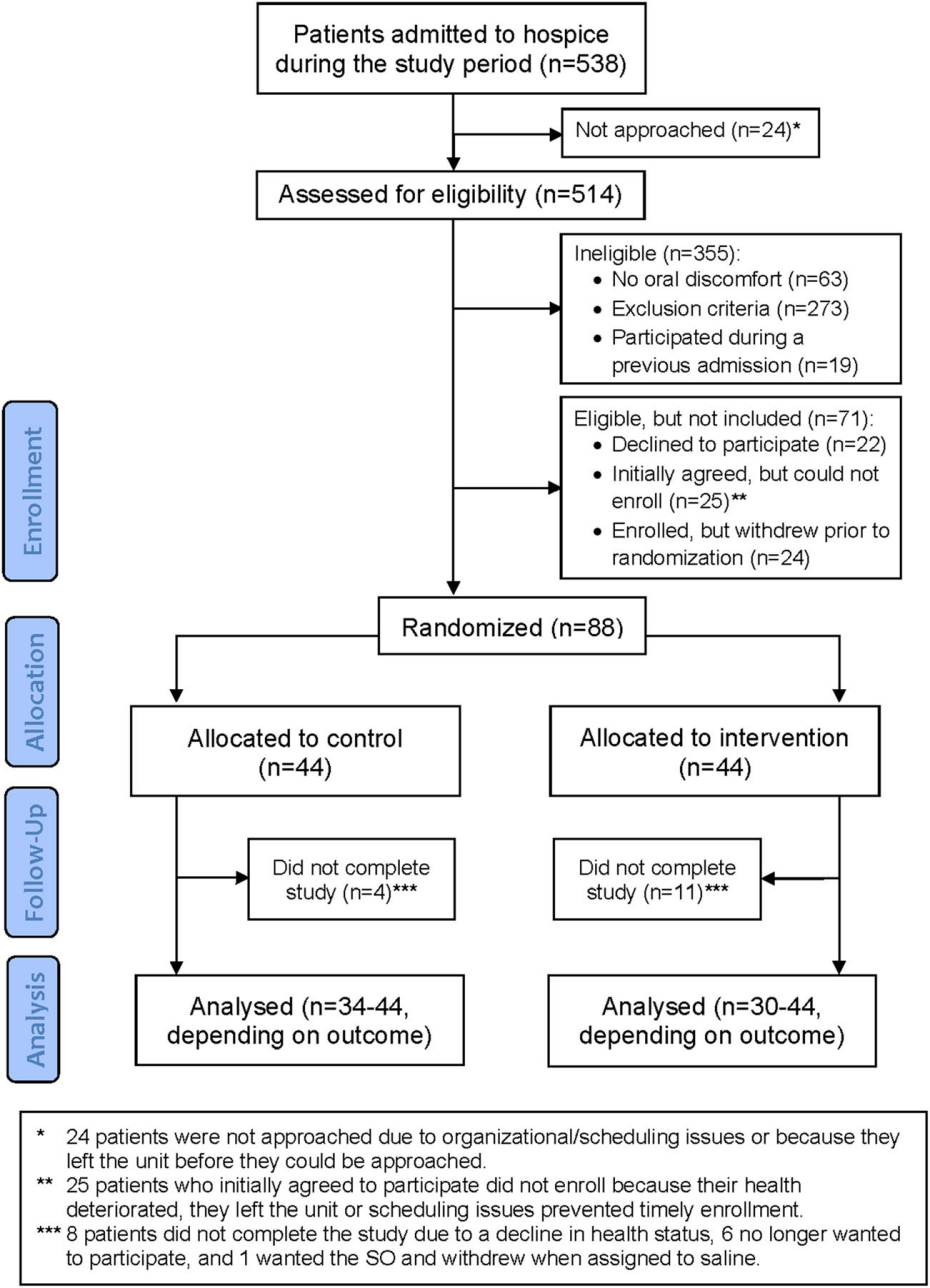
Participant flow chart

Of the 538 patients admitted to the hospice unit during the study period, 514 (96%) were evaluated for eligibility (Fig. 1).

### Variables and measures

The patients’ demographic and medical characteristics were obtained from their medical records. In order to describe the study sample, Karnofsky Performance Status (KPS) was used as a measure of functional performance, with scores ranging from 0 (death) to 100 (perfect health) [27]. Patients reported on their current oral care, including their frequency of tooth brushing and types of equipment used, like soft toothbrush, toothpaste, mouth rinse, floss, interdental brush, toothpick, and tongue cleaner.

The primary outcome for this study was the patient-reported outcome of oral symptoms, which were assessed on study days 1 and 5 to obtain measures before and after the 4-day intervention. Outcome data were collected by a nurse and a dentist who were blinded to patients’ treatment conditions and the mouth rinse used. The patient-reported outcome was assessed by using 12 items (#31–#42) selected from the European Organisation for Research and Treatment of Cancer (EORTC) Quality of Life Questionnaire–Oral Health 17 Phase III version (EORTC QLQ-OH17) [28], which is a supplementary module to the core questionnaire (EORTC QLQ-C30) [29]. At the time data collection was planned, the last version of the EORTC QLQ-OH15 Phase IV was not yet available [30]. The EORTC QLQ-OH17 measures oral problems and symptoms during the past week using a 4-point scale (1=not at all, 2=a little, 3=quite a bit, and 4=very much). Because the intervention lasted only 4 days, the timeframe was modified from “during the past week” to “during the past day.” Due to the challenges of conducting research in palliative care and to reduce participant burden, we also decided to use only the most relevant questions from the EORTC QLQ-OH17 module as single symptom items.

In addition, the patients’ daily experiences of (1) oral pain, (2) mouth dryness, (3) difficulty swallowing, and (4) pain while swallowing were assessed using a numerical rating scale (NRS) ranging from 0 (no discomfort) to 10 (maximum discomfort). Scores 1–3 indicate mild discomfort, 4–6 moderate discomfort, and 7–10 severe discomfort. NRS items were assessed between 12:00 and 14:00 each study day. On day 5, upon intervention completion, patients also answered the question: “What do you think about the mouth rinse?” using a NRS ranging from 0 (pleasant experience) to 10 (unpleasant experience). The use of the daily NRS allowed for the assessment of patients’ day-to-day experiences of the most common and/or distressing oral symptoms.

The secondary outcome was an objective assessment of oral health based on a clinical examination performed by a dentist before and after the intervention (days 1 and 5).

The oral examination included the dentist’s assessment of oral dryness, plaque on the teeth and tongue, the oral mucosa, the total number of teeth, and use of dentures and root remnants. Clinical evaluation of oral dryness was assessed by the sliding mirror test, drawing a dental mirror along the buccal mucosa, scored no/yes. When the mirror slid easily along the mucosa with no friction, it indicated no oral dryness, while friction indicated oral dryness [31]. Plaque on teeth was scored by using the plaque score index of the Mucosal-Plaque Score (MPS) assessment tool published by Henriksen and coworkers [32]. It rates the plaque from 1 to 4 (1=no easily visible plaque, 2=small amounts of hardly visible plaque, 3=moderate amounts of plaque, and 4=abundant amounts of confluent plaque). Plaque on tongue was scored using the same rating 1–4. Oral mucosal inflammation was scored by Oral Mucositis Assessment Scale (OMAS) [33], a detailed assessment tool for signs of mucositis: ulceration and erythema. Ulceration was scored 0–3, where 0=no lesion, 1= <1 cm^2^, 2= 1–3 cm^2^, and 3= >3 cm^2^. Erythema was scored 0–2, where 0=none, 1=mild, and 2=severe.

### Procedure and intervention

Both the intervention and control groups followed the same standardized procedure, which included basic oral care with tooth brushing morning and evening, rinsing with the assigned solution (10–15 ml) twice for 30 s four times a day, and after each rinsing, oral gel (1 cm) and lip balm were applied. In all patients, the same oral care products were used, such as a soft toothbrush (Jordan® Gum Protector), toothpaste (Biotene®), oral gel (Biotene®), and lip balm (Biotene®). The procedures were carried out four times daily, except for tooth brushing, which was performed every morning and evening. Patients could use the assigned rinse as needed in between the four daily intervention procedures.

The control group rinsed with NS and the intervention group with a SO solution consisting of 2.5 g SO herbal tea/100 ml water. The SO herbal tea solution was based on dry extract of *Salvia officinalis* leaves from the Hospital Pharmacy (Sanivo Pharma AS), which were steeped for 2 min in boiled water. The mouth rinses were prepared and replaced every morning during the intervention period and stored at room temperature, available for the patient as needed. Each rinsing (including extra rinses) was noted in the patient’s study journal. When required, nursing staff assisted patients with the rinsing procedure.

### Data analysis

Data were analyzed using SPSS 24.0 for Windows (IBM Corp, Armonk, NY). Descriptive statistics summarized demographic, medical, and mouth care variables. Continuous variables were presented as means and standard deviations (SD), and categorical variables as numbers and percentages. Chi-square tests and Fisher’s exact tests were used to compare groups on categorical variables. McNemar’s test was used to evaluate within-group change on categorical variables. Independent sample *t* tests were used to compare groups on continuous variables at specific timepoints, and paired *t* tests were used to evaluate within-group change on continuous variables. Linear mixed models were used to evaluate group differences over time, as indicated by significant group-by-day interactions. Analyses were conducted based on intention-to-treat. *p* values <0.05 were considered statistically significant. Cohen’s *d* ≥0.40 was considered a clinically significant effect size [34].

A power analysis indicated that a sample size of 88 (44 in each group) was sufficient to detect group differences over time (interaction) with a moderate effect size of *f*=0.25, assuming correlations of *r*=0.35 between repeated measures [35], a significance level of 5% and statistical power of 80%.

## Results

### Sample characteristics

Sample characteristics for the 88 enrolled patients are summarized in Table 1. There were no significant group differences at baseline, except that use of opiate medication was more frequent in the intervention group than the control group. There was no significant group difference on any measure of dental status (Table 1). At baseline, nearly all patients (90%) reported brushing their teeth twice or more daily, 41% used toothpicks and 29% used a mouth rinse. Of the 88 enrolled patients, 15 (17%) dropped out before completing the study, 4 in the control group, and 11 in the intervention group (*p*=0.087) (Fig. 1).

**Table 1:** Sample demographic and clinical characteristics

	Total sample (*n*=88)	Control (*n*=44)	Intervention (*n*=44)	*p* value
Age, years				0.728
Mean (SD)	63.9 (10.6)	63.5 (11.8)	64.3 (9.4)	
Range	29–84	29–84	45–83	
Sex, % (*n*)				0.151
Male	27% (24)	20% (9)	34% (15)	
Female	73% (64)	80% (35)	66% (29)	
Smoker	(*n*=83)	(*n*=42)	(*n*=41)	0.748
Yes, % (*n*)	23% (19)	21% (9)	24% (10)	
BMI	(*n*=76)	(*n*=38)	(*n*=38)	0.811
Mean (SD)	22.2 (3.8)	22.3 (4.1)	22.1 (3.6)	
Range	13.6–32.2	16.4–32.2	13.6–28.2	
Karnofsky score	(*n*=83)	(*n*=43)	(*n*=40)	0.504
Mean (SD)	52.1 (16.9)	53.3 (18.4)	50.8 (17.2)	
Range	20–80	20–80	20–80	
Primary diagnosis, % (n)				0.325^a^
Gastrointestinal cancer	26% (23)	29% (13)	23% (10)	
Lung cancer	17% (15)	16% (7)	18% (8)	
Gynecologic cancer	16% (14)	23% (10)	9% (4)	
Prostate cancer	3% (3)	5% (2)	2% (1)	
Breast cancer	13% (11)	9% (4)	16% (7)	
Other cancer	25% (22)	18% (8)	32% (14)	
Head/neck	8% (7)	7% (3)	9% (4)	
Metastases	(*n*=87)	(*n*=44)	(*n*=43)	
Yes, % (*n*)	83% (72)	82% (36)	84% (36)	0.814
Number of medications	(*n*=85)	(*n*=44)	(*n*=41)	0.161
Mean (SD)	11.4 (4.2)	10.8 (4.6)	12.1 (3.6)	
Range	4–26	4–26	4–20	
Type of medical treatment, % (n)	(*n*=85)	(*n*=44)	(*n*=41)	
Steroids	58% (49)	50% (22)	66% (27)	0.188
Opiates	85% (72)	77% (34)	93% (38)	**0.049**
Anti-depressants	24% (20)	20% (9)	27% (11)	0.489
Blood pressure medication	21% (18)	16% (7)	27% (11)	0.218
Paracetamol	59% (50)	57% (25)	61% (25)	0.697
Cardiac medication	22% (19)	25% (11)	20% (8)	0.544
Bisphosphonate therapy	7% (6)	5% (2)	10% (4)	0.427
Cancer treatment, % (n)				
Previous radiation therapy	55% (47)	57% (25)	52% (22)	0.679
on head/neck	16% (14)	18% (8)	14% (6)	0.560
Previous chemotherapy	86% (74)	84% (37)	88% (37)	0.592
Current chemotherapy	33% (28)	36% (16)	29% (12)	0.487
Dental status, mean (SD)	(*n*=84–86)	(*n*=43–44)	(*n*=41–42)	
Teeth count	23.6 (6.8)	23.8 (6.7)	23.4 (6.9)	0.803
Dentures count	0.4 (1.5)	0.3 (0.7)	0.5 (2.1)	0.144
Root remnant count	0.2 (0.6)	0.1 (0.3)	0.3 (0.8)	0.142

^a^Fisher’s exact test; ^b^*n*=80 for total sample, *n*=43 for control, and *n*=37 for intervention

### Baseline symptoms of oral discomfort

**Fig. 2:**
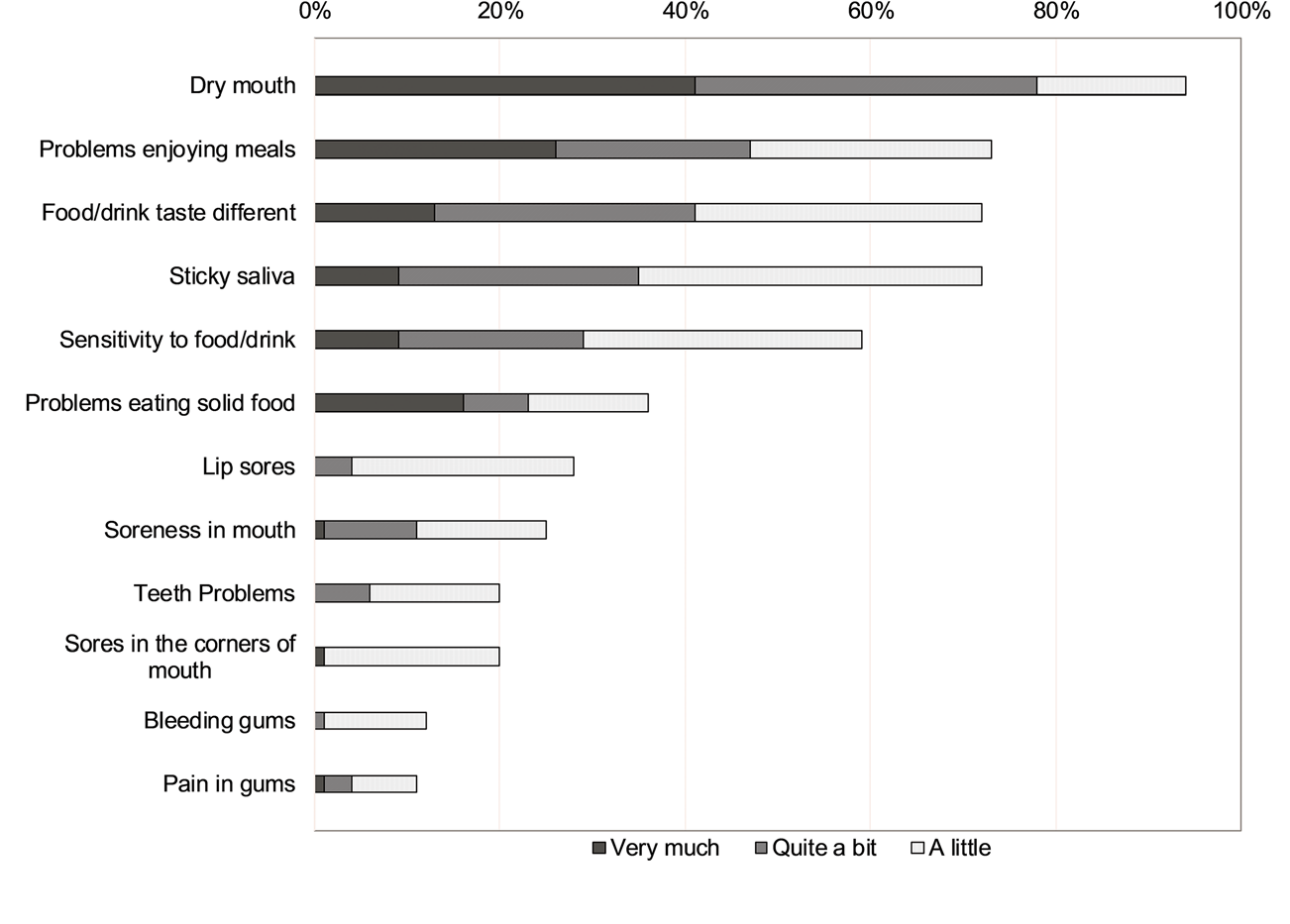
Patient-reported oral symptoms at baseline (12 items from the EORTC QLQ-OH17)

Of the 12 oral symptoms assessed using items from the EORTC QLQ-OH17, the most common and severe symptom was dry mouth, with 78% of patients experiencing oral dryness either “quite a bit” or “very much” (Fig. 2). Other common symptoms of oral discomfort included problems enjoying meals, food and drink tasting different, and sticky saliva, with >70% of patients reporting these symptoms at least “a little”.

On baseline NRS ratings of oral discomfort, 94% of patients reported some dry mouth (rating>0) and 80% reported moderate to severe dry mouth (rating ≥4), with no difference between groups (*p*=0.872). About half of the patients (48%) reported some difficulty swallowing and 24% reported moderate to severe difficulty swallowing, again with no significant difference between groups (*p*=0.109). Because <20% of patients reported any pain in their mouth or when swallowing, and <8% rated them ≥4 on the NRS, these symptoms were not included in subsequent analyses.

Baseline clinical evaluations indicated that most patients had at least small amounts of hardly visible plaque on both the tongue (67%) and teeth (58%). Fewer than 10% had abundant amounts of confluent dental plaque. In addition, at baseline, 47% of patients had a positive sliding mirror test indicating oral dryness. On the baseline OMAS scoring, 76% of patients had at least some evidence of erythema (score >0), but only 5% had scores >1. With respect to ulceration, 45% had no evidence of ulceration, and only 4% had scores >0.5.

### Evaluation of intervention effects

**Fig. 3:**
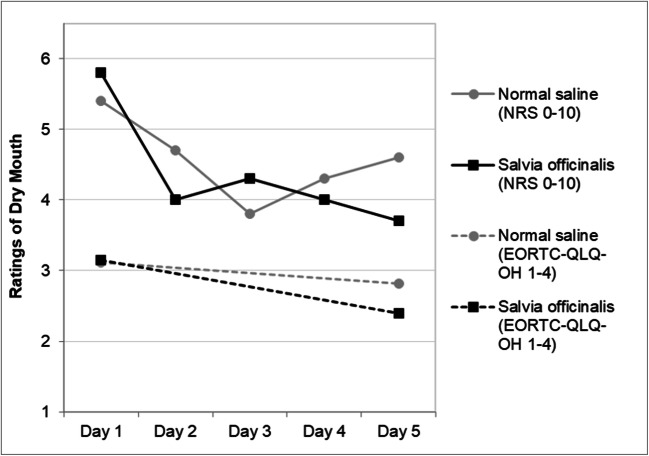
Changes in EORTC QLQ-OH17 and NRS ratings based on dry mouth by treatment group (group-by-time interaction *p*=0.036 and 0.045, respectively). For the EORTC QLQ-OH17, the effect size for the difference between groups on day 5 is Cohen’s *d*=0.45; the effect size for improvement over time was *d*=0.33 for the saline group and 0.75 for the salvia group. For the NRS, the effect size for the difference between groups on day 5 is Cohen’s *d*=0.31; the effect size for improvement over time was *d*=0.39 for the saline group, and 0.63 for the salvia group

The normal saline and *Salvia officinalis* rinses resulted in similarly improved oral comfort mean scores on the 12 items from the EORTC QLQ-OH17 between study days 1 and 5, (*p*=0.001 and *p*=0.003, respectively) (Table 2). With respect to individual symptoms, both the NS and SO rinses resulted in improved ratings of dry mouth on EORTC QLQ-OH17 item #37 and the NRS between days 1 and 5, but the reported improvements were significantly higher in the SO group than in the NS group, as evidenced by the significant group-by-day interaction (Fig. 3).

**Table 2:** Change in patient-reported outcomes between days 1 and 5 by treatment group

	Control (*n*=44)	Intervention (*n*=44)	*p* value (*d* value) for group *t* test	*p* values for mixed model effects
EORTC QLQ-OH17 (1-4 scale)				
12-item mean score				GxD p=0.889; day *p*<**0.001**
Day 1	1.80 (0.37) (*n*=43)	1.72 (0.39) (*n*=40)	*p*=0.305	
Day 5	1.62 (0.31) (*n*=39)	1.50 (0.40) (*n*=33)	*p*=0.149	
Day paired *t* test	*p*=**0.001** (*n*=39; *d*=0.58)	*p*=**0.003** (*n*=33, *d*=0.56)		
#35 mouth soreness				GxD *p*=0.462; day *p*=0.302
Day 1	1.42 (0.73) (*n*=43)	1.33 (0.69) (*n*=40)	*p*=0.552	
Day 5	1.26 (0.72) (*n*=39)	1.27 (0.67) (*n*=33)	*p*=0.921	
Day paired *t* test	*p*=0.173 (*n*=39)	*p*=0.801 (*n*=33)		
#36 scores in corners of mouth				GxD *p*=0.738; day *p*=**0.024**
Day 1	1.28 (0.59) (*n*=43)	1.18 (0.39) (*n*=40)	*p*=0.348	
Day 5	1.21 (0.47) (*n*=39)	1.06 (0.24) (*n*=33)	*p*=0.099*	
Day paired *t* test	*p*=0.160 (*n*=39, *d*=0.23)	*p*=**0.044** (*n*=33, *d*=0.37)		
#37 dry mouth				GxD *p*=**0.036**; day *p*<**0.001**
Day 1	3.12 (0.80) (*n*=42)	3.15 (1.00) (*n*=40)	*p*=0.878	
Day 5	2.82 (0.91) (*n*=39)	2.39 (1.00) (*n*=33)	*p*=0.063 (*d*=0.45)	
Day paired *t* test	*p*=**0.048** (*n*=38, *d*=0.33)	*p*<**0.001** (*n*=33; *d*=0.75)		
#38 sticky saliva				GxD *p*=0.347; day *p*<**0.001**
Day 1	2.07 (0.89) (*n*=42)	2.23 (0.99) (*n*=39)	*p*=0.448	
Day 5	1.76 (0.82) (*n*=38)	1.76 (0.87) (*n*=33)	*p*=0.978	
Day paired *t* test	*p*=0.077 (*n*=38, *d*=0.30)	*p*=**0.001** (*n*=33, *d*=0.64)		
#39 food/drink sensitivity				GxD *p*=0.490; day *p*=0.080
Day 1	2.10 (1.09) (*n*=41)	1.80 (0.85) (*n*=40)	*p*=0.176	
Day 5	1.83 (1.03) (*n*=36)	1.64 (0.86) (*n*=33)	*p*=0.393	
Day paired *t* test	*p*=0.091 (*n*=34, *d*=0.30)	*p*=0.500 (*n*=33, *d*=0.12)		
#40 taste different				GxD *p*=0.760; day *p*=**0.021**
Day 1	2.48 (1.07) (*n*=42)	2.05 (0.93) (*n*=40)	*p*=0.058	
Day 5	2.24 (1.15) (*n*=38)	1.75 (0.88) (*n*=32)	*p*=**0.049** (*d*=0.48)	
Day paired *t* test	*p*=**0.046** (*n*=37, *d*=0.34)	*p*=0.206 (*n*=32, *d*=0.23)		
#41 problems eating solid food				GxD *p*=0.689; day *p*=**0.002**
Day 1	1.65 (1.02) (*n*=43)	1.85 (1.25) (*n*=40)	*p*=0.429	
Day 5	1.36 (0.87) (*n*=39)	1.52 (0.94) (*n*=33)	*p*=0.468	
Day paired *t* test	*p*=**0.008** (*n*=39, *d*=0.45)	*p*=0.206 (*n*=33, *d*=0.23)		
#42 problems enjoying meals				GxD *p*=0.401, day *p*=0.051
Day 1	2.58 (1.12) (*n*=43)	2.28 (1.19) (*n*=39)	*p*=0.244	
Day 5	2.49 (1.25) (*n*=39)	1.85 (1.06) (*n*=33)	*p*=**0.022** (*d*=0.55)	
Day paired *t* test	*p*=0.313 (*n*=39, *d*=0.16)	*p*=0.096 (*n*=32, *d*=0.30)		
Patient perception of oral discomfort (0-10 NRS)				
Mouth dryness				GxD *p*=**0.045**; day *p*<**0.001**
Day 1	5.4 (2.3) (*n*=43)	5.8 (3.0) (*n*=41)	*p*=0.560	
Day 2	4.7 (2.6) (*n*=41)	4.0 (3.1) (*n*=39)	*p*=0.270	
Day 3	3.8 (2.6) (*n*=41)	4.3 (3.0) (*n*=38)	*p*=0.440	
Day 4	4.3 (3.2) (*n*=39)	4.0 (3.0) (*n*=35)	*p*=0.727	
Day 5	4.6 (2.7) (*n*=40)	3.7 (3.0) (*n*=33)	*p*=0.187	
Day 1–5 paired *t* test	*p*=**0.017** (*n*=40, *d*=0.39)	*p*=**0.001** (*n*=33, *d*=0.63)		
Difficulty swallowing				GxD *p*=0.756; day *p*=**0.013**
Day 1	1.4 (2.4) (*n*=42)	2.2 (2.3) (*n*=41)	*p*=0.144	
Day 2	1.0 (1.7) (*n*=41)	1.6 (2.4) (*n*=39)	*p*=0.283*	
Day 3	0.7 (1.5) (*n*=41)	1.3 (2.0) (*n*=38)	*p*=0.170*	
Day 4	0.9 (1.6) (*n*=39)	1.5 (2.1) (*n*=35)	*p*=0.150	
Day 5	0.9 (1.5) (*n*=40)	1.6 (2.6) (*n*=33)	*p*=0.183	
Day 1–5 paired *t* test	*p*=0.091 (*n*=39, *d*=0.28)	*p*=0.549 (*n*=33, *d*=0.11)		

*GxD* group by day interaction effect. *p* values <0.05 are bolded. Cohen’s *d* is reported for within group *t* tests when *p*<0.10 for either treatment group and is reported for day 5 between-group comparisons when *p*<0.05

*Separate variance *t* test was used due to unequal variances

Clinical evaluation showed significantly improved OMAS erythema scores between days 1 and 5 in the NS group only (Table 3). Significantly reduced plaque on teeth was registered both in the NS and SO groups between days 1 and 5. For plaque on the tongue, both groups showed similar improvement in scores, but only the NS group reached statistical significance. There was no significant difference in patients’ experience of the mouth rinses between the NS (mean 3.45, SD 2.86) and SO groups (mean 2.73, SD 2.76, *p*=0.72), with higher scores indicating less pleasant experiences.

**Table 3:** Change in OMAS and clinical evaluation between days 1 and 5 by treatment group

	Control (*n*=44)	Intervention (*n*=44)	*p* value for group *t* test	*p* values for mixed model effects
OMAS				
Ulceration score (0–3)				GxD *p*=0.706; day *p*=0.378
Day 1	0.12 (0.21) (*n*=44)	0.13 (0.29) (*n*=42)	*p*=0.837	
Day 5	0.09 (0.17) (*n*=40)	0.10 (0.23) (*n*=33)	*p*=0.750	
Day paired *t* test	*p*=0.083 (*n*=40, *d*=0.28)	*p*=0.800 (*n*=33, *d*=-0.04)		
Erythema score (0–2)				GxD *p*=0.481; day *p*=**0.022**
Day 1	0.40 (0.39) (*n*=44)	0.34 (0.30) (*n*=42)	*p*=0.491	
Day 5	0.31 (0.35) (*n*=40)	0.27 (0.24) (*n*=33)	*p*=0.506	
Day paired *t* test	*p*=**0.022** (*n*=40, *d*=0.38)	*p*=0.498 (*n*=33, *d*=0.12)		
Clinical evaluation				
Plaque on tongue (1–4)				GxD *p*=0.854; day *p*=**0.003**
Day 1	2.02 (0.74) (*n*=43)	2.07 (1.11) (*n*=42)	*p*=0.815	
Day 5	1.70 (0.79) (*n*=40)	1.76 (0.75) (*n*=33)	*p*=0.752	
Day paired *t* test	*p*=**0.018** (*n*=39, *d*=0.40)	*p*=0.083 (*n*=33, *d*=0.31)		
Plaque on teeth (1–4)				GxD *p*=0.279; day *p*<**0.001**
Day 1	1.66 (0.68) (*n*=44)	2.00 (0.95) (*n*=41)	*p*=0.059	
Day 5	1.45 (0.60) (*n*=40)	1.61 (0.61) (*n*=33)	*p*=0.274	
Day paired *t* test	*p*=**0.018** (*n*=40, *d*=0.39)	*p*=**0.008** (*n*=33, *d*=0.49)		
Sign of oral dryness				GxD *p*=0.385; day *p*=**0.001**
Day 1	45% (18/40)	49% (19/39)	*p*=0.741	
Day 5	20% (7/35)	32% (10/31)	*p*=0.256	
Day McNemar test	*p*=**0.022** (*n*=35)	*p*=0.125 (*n*=30)		

*GxD* group by day interaction effect. *p* values <0.05 are bolded. Cohen’s *d* is reported for within group *t* tests when *p*<0.10 for either treatment group

## Discussion

The present study investigated the effect of a mouth rinse based on the herb *Salvia officinalis* (SO) compared with standard care using normal saline (NS) as a mouth rinse among hospice patients with late-stage cancer. Our findings showed significant improvement in both groups on patient-reported oral symptoms and on clinical evaluation measures after 4 days of intervention. There were no overall statistically significant group differences between the SO and saline mouth rinses. Given the palliative care setting, the intervention timeframe was 4 days, which may have limited the observed effects.

In our study sample, the most prevalent patient-reported oral symptom at baseline was dry mouth, with 78% reporting significant oral dryness on the EORTC QLQ-OH17 and 80% reporting moderate to severe dry mouth on the NRS. This result is consistent with other studies in similar populations, where dry mouth has been reported in 77–78% of terminally ill cancer patients [1, 8, 36, 37], with medication being the major cause [15]. In this study, patients were taking an average of 11 medications and 85% used opiates. The only group difference at baseline was in the use of opiates, 77% in the NS group and 93% in the SO group (*p*=0.049). Despite this difference in use of opiates, no difference was found regarding presence of xerostomia at baseline. Anti-cancer treatment, such as chemotherapy, may cause xerostomia [4, 5], and 33% of the patients in this study were currently on palliative chemotherapy. However, there were no group differences at baseline with respect to type of chemotherapy or degree of xerostomia.

Although there was no significant difference in patient-reported symptom scores in xerostomia between the groups after 4 days of intervention, there was a significant improvement over time within both groups. In addition, the effect size of the reduction in dry mouth over time was in the medium-to-large range for the SO group and in the small-to-medium range for the NS group. Both rinses used in the present study had a positive effect on patients’ subjective experience of xerostomia and could therefore be recommended for palliation of oral discomfort.

Dysgeusia, or taste alteration, is a frequently reported oral symptom in palliative care patients, with a prevalence of 60–80% [38]. The prevalence of taste alteration among the patients in this study was similar to prior estimates, with more than 70% reporting that food/drinks tasted at least “a little” different (Fig. 2). Taste alteration may also impact patients’ daily quality of life, food intake, and dysphagia, as well as be a symptom of oral fungal infection [38, 39]. Given its prevalence and potential impact on other areas of function, taste alteration should be included in any assessment of oral symptoms.

At baseline, 90% of patients reported brushing their teeth twice daily or more. Despite good oral hygiene and dental status, clinical evaluation indicated that 76% had evidence of mild erythema. In a prior study with terminally ill cancer patients, 50% had erythema assessed by the OMAS [7]. Although patients on or in need of antifungal treatment based on clinical signs were excluded from our study, oral fungal infection among cancer patients in palliative care may occur with either asymptomatic and symptomatic features [40]. Erythema reported in the current study could thus be related to ongoing or previous chemotherapy, oral dryness, and/or an asymptomatic oral fungal infection. At baseline, none of the patients was being treated with antifungal medication nor did they show obvious clinical signs of oral candidiasis. However, a swab test for *Candida* carriage was taken at baseline of all patients and further fungal analyses will be presented in another study.

This study followed the procedures published at ClinicalTrials.gov, but has several limitations. Most importantly, the observed improvement in the two groups is likely not due to the mouth rinse alone, but is also likely the result of the structured mouth care regimen, of which regular rinsing was only part. Moreover, most of the patients were also using lubricant products, even at baseline. Given that 80% of patients with advanced cancer report dry mouth and these products are routinely used in the treatment of dry mouth, it was considered unethical to request that patients stop using them. Because some of the participants were in need of oral lubricant, the oral care protocol was standardized to include use of oral gel and lip balm in order to minimize group differences. Additionally, due to SO’s distinctive taste and smell, patients were not blinded to their group assignment.

Although patients did not compare the SO and NS rinses with each other, both rinses received ratings indicating that patients were generally satisfied with the one they received. Both SO and NS are inexpensive rinses, and being alcohol-free, they are likely to be gentle to the mucosa in the oral cavity. However, the SO mouth rinse is also drinkable, and therefore harmless for patients with swallowing disorders and problems with spitting out the mouth rinse.

Clinical studies in palliative care must utilize procedures that are as minimally burdensome as possible to the patients. Questionnaires must be brief and easy to answer and clinical examinations must be kept as simple as possible [41]. This was the reason for excluding items from the EORTC QLQ-OH17 questionnaire, as some items (#43–#47) were considered less applicable to our study group given the patients’ advanced disease and situation. The study procedure was designed with consideration of these challenges, but future studies in this area would be strengthened by addressing these limitations. Lastly, it is worth noting that only 22 patients (14%) declined to participate, although an additional 24 patients initially agreed, but later declined. This shows that patients at end-of-life often want to contribute to clinical trials despite their fragile situation.

### Conclusion

The results of this study indicate that the *Salvia officinalis* mouth rinse intervention was as good as, but not significantly better than normal saline rinsing for reducing oral symptoms, particularly dry mouth among cancer patients in palliative care. Rinsing four times a day with either solution, together with a basic oral care program, improved patients’ oral status and reduced oral discomfort. It would be a considerable improvement for palliative care units to collaborate with and include oral health professionals to identify and manage oral health issues in order to achieve the best patient care and quality of life for these patients.
